# Clear obstacles and hidden challenges: understanding recruiter perspectives in six pragmatic randomised controlled trials

**DOI:** 10.1186/1745-6215-15-5

**Published:** 2014-01-06

**Authors:** Jenny L Donovan, Sangeetha Paramasivan, Isabel de Salis, Merran Toerien

**Affiliations:** 1 School of Social and Community Medicine, University of Bristol, Canynge Hall, Bristol BS8 2PS, UK; 2 Department of Sociology, University of York, Heslington, York YO10 5DD, UK

**Keywords:** Equipoise, Qualitative research, Randomised controlled trials, Recruitment

## Abstract

**Background:**

Recruitment of sufficient participants in an efficient manner is still widely acknowledged to be a major challenge to the mounting and completion of randomised controlled trials (RCTs). Few recruitment interventions have involved staff undertaking recruitment. This study aimed i) to understand the recruitment process from the perspective of recruiters actively recruiting RCT participants in six pragmatic RCTs, and ii) to identify opportunities for interventions to improve recruitment.

**Methods:**

Interviews were undertaken with 72 individuals (32 doctors or RCT Chief investigators (CIs); 40 nurses/other health professionals) who were actively recruiting participants in six RCTs to explore their experiences of recruitment. The RCTs varied in scale, duration, and clinical contexts. Interviews were fully transcribed and analysed using qualitative content and thematic analytic methods derived from grounded theory. For this analysis, data were systematically extracted from each RCT and synthesised across all six RCTs to produce a detailed and nuanced understanding of the recruitment process from the perspectives of the recruiters.

**Results:**

Recruiters readily identified organisational difficulties, fewer than expected eligible patients, and patients’ treatment preferences as the key barriers to recruitment. As they described their experiences of recruitment, several previously hidden issues related to their roles as researchers and clinicians emerged, imbued with discomfort and emotion. The synthesis across the RCTs showed that doctors were uncomfortable about aspects of patient eligibility and the effectiveness of interventions, whereas nurses were anxious about approaching potential RCT participants and conflicts between the research and their clinical responsibilities. Recruiters seemed unaware that their views contributed to recruitment difficulties. Their views were not known to RCT CIs. Training and support needs were identified for both groups of staff.

**Conclusions:**

The synthesis showed that recruitment to these RCTs was a complex and fragile process. Clear obstacles were identified but hidden challenges related to recruiters’ roles undermined recruitment, unbeknown to RCT CIs. Qualitative research can elicit and identify the hidden challenges. Training and support are then needed for recruiters to become more comfortable with the design and principles of RCTs, so that they can engage more openly with potentially eligible participants and create a more resilient recruitment process.

## Background

Recruitment of sufficient eligible participants is widely acknowledged to be a major challenge to randomised controlled trials (RCTs), leading to underpowered studies, additional resources spent on costly extensions and, in some cases, the inability to tackle key healthcare questions [1,2]. Several systematic reviews have identified particular issues that RCTs need to address in order to recruit successfully, including that the RCT must tackle an important and timely question, with a clear protocol and straightforward and low impact data collection [2-4], employ dedicated research staff [2,3], ensure prior planning and piloting of recruitment [2,3], ensure good communication and training for staff about trial processes and interventions [2,5], and use proven effective recruitment strategies [6]. These sensible and practical suggestions are mostly drawn from the experience of trialists and from individual RCTs; only a very small number of robust interventions have been shown to improve recruitment, including telephone reminders or financial inducements [7], newsletters and mail-shots to encourage non-responders [2], and opt-out rather than opt-in schemes for participants [6].

Recruitment is an interactional activity and typically occurs after at least two meetings between a potential participant and clinical and/or researcher staff, initially to establish whether the participant is eligible for the RCT according to the formal criteria in the protocol, and then, if they are, the provision of information about the details of the RCT and discussion about a decision to participate, concluded by the signing of a consent form. The length and complexity of this process is in sharp contrast with the tendency of the interventions above to focus on particular practical matters. It is also remarkable that systematic reviews have consistently indicated the importance of recruiters in RCT recruitment, but there is still little understanding of the perspective of recruiters and very few interventions that have been targeted at recruitment staff [6]. Few studies have even considered how the process of recruitment occurs, or the influence that recruiters might have on it. A study using qualitative research methods integrated in one RCT developed a complex intervention based on an understanding of the recruitment process from the perspective of recruiters [8]. Attempts were then made to apply the intervention to other RCTs, but a number of difficulties were experienced [9].

This paper seeks to build on that qualitative research to produce a detailed understanding of the recruitment process from the perspective of active recruiters through an investigation of recruitment in six independent, pragmatic, publicly-funded RCTs with complex interventions ranging across a number of clinical centres and contexts. The RCTs were either experiencing severe recruitment problems, or were pilot/feasibility studies expecting difficulties, and each had included or added an embedded qualitative study to understand the recruitment process, particularly from the perspective of recruiters, based on an intervention developed in the first RCT studied [8]. Data were collected in each of the six RCTs from recruitment staff (doctors, nurses, and researchers) in interviews while they were actively trying to recruit RCT participants and in this article these data have been synthesised across the RCTs to produce a detailed and nuanced understanding of recruitment by recruiters. Many barriers to recruitment were identified. Often, as suggested from previous research, these seemed to be related to organisational challenges or the discovery that large numbers of eligible patients had strong preferences for particular interventions. As recruiters reflected on their experiences of recruitment in the interviews, a number of other underlying and somewhat hidden issues emerged, associated with their views about research and their roles in research and clinical care. The findings exposed issues underlying the fragility of RCT recruitment, and, in turn, enabled the identification of opportunities to develop more resilient and effective recruitment processes that should improve efficiency as well as experiences for recruiters and patients.

## Methods

The research was undertaken in six publicly-funded RCTs in a range of clinical contexts, with different types of RCT intervention, with a range of primary recruiters, and at different stages of the implementation of the RCT (Table 1) [9]. Qualitative research methods were used to ensure detailed exploration of the perspectives of recruitment by active recruitment staff and some RCT methodologists. In-depth interviews were conducted in each of the RCTs, using a checklist of topics to ensure that similar issues were covered whilst enabling other issues of importance to emerge. Interviews were conducted by ZT, SP, IdS, MT, or GDW, mostly face-to-face and one-to-one. Opportunities were taken on six occasions to discuss emerging issues with groups when they met as part of the conduct of the RCT. In four RCTs, there was sufficient time for follow-up interviews to be carried out amongst a subsample, enabling some recruiters to reflect on the issues raised by the studies and the feedback they had received. Consent was obtained for interviews and discussions to be audio-recorded. Ethical approval was obtained from the bodies listed below (additional information).

**Table 1:** RCT characteristics and numbers of study participants and interviews

**RCT code**	**Clinical context**	**RCT type**	**Clinical centres**	**Interventions**	**Specialties involved**	**Primary recruiters**	**Doctors, CIs or TMG**	**Nurses**	**Follow-up interviews**	**Total number of interviews**
			**n**				**n**	**n**	**n**	**n**
T1	Cancer	Feasibility	5	Surgery or radiotherapy	Surgery and oncology	Doctors and nurses	3		3 (1 group)	**6**
T2	Primary care and cancer	Main	28	Multiple follow-up strategies	Primary care and oncology	Doctors and nurses	3	3	3 (1 group)	**9**
T3	Primary care and infection	Main	1 area (multiple locations)	Drug x, drug y, drug x + y	Primary care and paediatrics	Nurses	2	2		**4**
T5	Mental health and community care	Main	2 areas (multiple locations)	Specific support or usual care	Psychiatry, community mental services	Nurses and others	3	22 (2 groups)	2 (1 group)	**27**
T6	Cancer	Feasibility and main	9	Surgery, radiotherapy, monitoring	Surgery and oncology	Doctors and nurses	13	10 (1 group)	7	**30**
T7	Cancer	Feasibility	22	Chemotherapy with surgery or with radiotherapy	Oncology and surgery	Doctors	8	3		**11**
**Total numbers of interviews**							**32**	**40**	**15**	**87**

CI: Chief investigator; TMG: Trial management group.

Interviews and discussions were transcribed in full and analysed using qualitative content and thematic analysis methods, based on the techniques of constant comparison from grounded theory [10,11]. The researchers, ZT, IdS, MT, and SP, coded the interview transcript data for each RCT separately using ATLAS-ti qualitative data analysis software. A basic coding framework was developed by the team and applied to the data from each RCT separately to identify themes relating to the process of recruitment [12]. A detailed descriptive account of the thematic findings was then produced, providing a summary and holistic (ethnographic) account of the recruitment process in each RCT. This was then used to provide anonymised feedback to the RCT chief investigator (CI) and develop information provision and training for recruiters [9]. For this manuscript, JLD first considered the summary accounts from each RCT to identify recruitment issues that were similar or different between the RCTs. This provided a framework, but for a full synthesis, the original data from the interview transcripts in all six RCTs were extracted and systematically coded by JLD to synthesise the themes across the RCTs, focusing on recruiters’ perceptions and experiences of the recruitment process, the barriers they identified, and the exploration of other issues that were inhibiting or facilitating recruitment. The findings of this synthesis are presented here.

## Results

### Characteristics of RCTs and participants

The six RCTs were funded by several major UK public funding bodies and their clinical contexts included cancer surgery, oncology, paediatrics, and psychiatry/mental health (Table 1). They included a variety of interventions including drugs, surgery, radiotherapy, and social support. Two of the RCTs (T1 and T7) were feasibility studies in which the CI anticipated recruitment difficulties; T2, T3, and T5 were full-scale RCTs where recruitment had not initially been expected to be problematic; and T6 included a feasibility phase where recruitment difficulties were anticipated and a main, successfully recruiting phase. Interviews were undertaken with 72 individuals who were actively recruiting participants (32 were doctors or RCT CIs, 40 were nurses or other health professionals). The doctors were all employed by the NHS and carried out eligibility assessments and recruitment in all but T5 (where this was done by clinical care coordinators) as part of their combined clinical and research roles. The nurses in T3 and T6 were employed by the RCT as research nurses, and T5 employed a researcher specifically to undertake recruitment. In contrast, T1, T2, and T7 relied primarily on research nurses employed by the National Cancer Research Network (NCRN), who were trained centrally to recruit patients to all available RCTs.

In each RCT, the CI and between one and three Trial Management Group (TMG) members were interviewed to provide an overview of the RCT, and a sample of active recruiters was also approached, including the principal investigators of clinical centres and other staff. There were 15 follow-up interviews in the RCTs where funding was available for this, seven with individuals in T6 and eight in groups in T1, T2, and T5. Most interviews (47) were one-to-one, but some were in groups (Table 1). The qualitative studies were added to the RCTs over an extended period of time, and so initial interviews were conducted in 2001/2002 in T6; in 2005 for T1, T2, and T3; 2006 for T5; and 2009 for T7. In T1, T2, T6, and T7, medically qualified and nursing staff were involved in recruitment; in T3 and T5, the primary recruiters were nurses or other staff.

In the presentation of the results below, RCTs and participants have been anonymized to protect confidentiality. Each staff member has been labelled according to the RCT (e.g., T7); whether they were a doctor, CI, or member of the TMG (e.g., –D9), a nurse or other health professional (−N7); and whether the interview was individual (no label), group (−G) or follow-up (−F).

### Understanding recruitment: recruiter perspective**s**

All recruiters readily described the barriers that they were convinced hindered recruitment. The clearest and most common barriers were organisational difficulties, fewer eligible patients than anticipated, and strong preferences expressed by patients for particular treatments or against being randomised:

T2–D1: “*The actual difficulty was in the logistics and it’s usually because the nurse can’t attend the clinic or the nurse can’t attend the meeting or the multidisciplinary team doesn’t actually go through the list of patients that are eligible at that time.*”

T1–D2: “*So, so there’s a local nuance there that we do get a significant number of patients who will plump for* [intervention 1] *if they live in* [district a]*. They don’t want to travel. They live next to the* [intervention 1] *centre which is I guess, what, ten miles difference or something like that, eight, ten miles? And conversely patients will plump for* [intervention 2] *for the fact that it’s going to happen in the hospital down the road.*”

T1–N2–FG: “*All the patients that have said ‘no’ , have said ‘no’ for personal reasons, not because of anything that we could change. One of them didn’t want a computer to decide what was happening about his treatment, he had to have control over his own destiny, and he just didn’t like the idea of being randomised.”*

These barriers were consistently reported and recruiters often seemed overwhelmed by the difficulties, leading to low morale and very poor recruitment levels:

T7–D5: *“I’m a genuine believer in the principle that [intervention 1] might be as good as* [intervention 2] *and therefore I’m a strong supporter of the basis of this trial … But it’s been a nightmare, frankly … I love this trial. I think it’s beautifully designed, I think it is extremely clever, which fits* [name of CI] *who is extremely clever … But, I honestly have never had as many problems with recruiting to a trial in all my born days.”*

As would be expected, the leaders of the RCTs tried hard to find solutions to these difficulties, by changing organisational arrangements of clinics, or making sure that all potentially eligible patients could be included:

T1–N2–FG: “*The patients get the diagnosis and they see the surgeon and the oncologist on the same day. They mention* [RCT] *to the patients and then if they want to go in it, they refer the patient to me then they come in to me sometime in the next week or so … They’ve had both treatments explained to them a couple of days ago by the time they get to me, so it’s quite good.*”

T7–D4: “[We are going to] *make certain that all potentially eligible patients have a discussion about the trial and know that it’s available for them … have a research nurse attending the MDT* [multi-disciplinary team meeting, where treatments are decided]. *In terms of the organisational things, it’s challenging, but it’s not impossible.*”

Some organisational changes slightly improved recruitment in some RCTs, but it remained problematic. Recruiters had been encouraged, in the interviews, to recount their actual experiences of recruitment in detail. As they did so, it became apparent that there were issues associated with their role as a recruiter that they found difficult, particularly in relation to their other roles as practising doctors or nurses. As they described their roles and the challenges and conflicts within them, they began to express emotion and discomfort, sometimes defensiveness, and a number of previously hidden challenges to recruitment emerged; these are detailed below.

### ‘Hidden’ challenges: complex roles

Recruiters had several roles, some perceived to be complementary, and others conflicting. While there were some similarities between some doctors and nurses, there was much more coherence within each of the professional groups and considerable differences between them, and so their views are presented separately below. While the recruiters eagerly described their roles and their challenges and expressed their views freely to the interviewer, very few linked their views or feelings with an impact on RCT recruitment or had raised these issues with the RCT leads.

### 1. Doctors’ roles

Doctors were involved in recruitment in all these RCTs, most in assessments of eligibility and sometimes undertaking the process of informed consent on their own or with the assistance of nurses or other researchers. When asked directly, these doctors described themselves as scientists or practising clinicians, or some combination of the two. Some were clear that these roles were complementary, and that it was really important to them to be able to combine the often urgent need to collect robust evidence through RCTs with a clinical role.

T3–D1: “*I see my role as a researcher and as a clinician as engaging my patients and the community out there in a long-term relationship, not just for today, not just for this child, not just for this illness, but a long-term relationship for us together, to improve clinical practice and reduce uncertainty. And that’s about saying to people ‘okay that’s fine this time you don’t want to do it perhaps another time you will, perhaps another time the condition will be the right one for you or the timing will be better’.*”

T6–D11: “*I’m convinced fully about what I’m doing … Unless we take this attitude of impartiality we will not find out and we will continue to over-treat patients or to under-treat patients.”*

When asked directly, the doctors were positive about their involvement in the RCT and their commitment to it, but then went on to describe a number of underlying conflicts with clinical practice that they experienced to varying degrees. These related particularly to the balance between carrying out research and ensuring that they safeguarded the best interests of the individual patient, and their willingness or ability to suspend their clinical judgments and recruit an individual patient to the RCT. In talking about the RCT, it was easy for them to agree that there was a lack of evidence about the most suitable treatment in general, but many admitted that when faced with a particular patient, or those with specific clinical characteristics, they became more uncomfortable:

T7–D2: “*There’s always a slight conflict between the patient sitting in front of you and their wishes but also taking part in the trial and wanting to support the trial, wanting to increase recruitment. Yes and it’s an element of frustration when someone says, ‘no’. So there can be a conflict between your management responsibilities for an individual compared to your wish to make something else a success.*”

T6–D2–F: “*Sometimes you get the feeling that we are counselling them to the extent that you are doing a disservice to them. Are you helping or hindering their management when you yourself know there are so many uncertainties and at the moment, or as far as you know, there are controversies, but all treatments can be similar with minute variations … So, it’s inevitable at that times you feel uncomfortable.*”

Some went on to describe particular patients or groups of patients who were technically eligible for the RCT, but who they felt were not really suitable and were either uncomfortable about recruiting, or definitely would not recruit:

T6–D12: “*I wouldn’t be involved with the trial because it wouldn’t be ethical if I had a strong view that this wasn’t right … But, there are a subset of younger men with more aggressive tumours where I find that really quite difficult.*”

T7–D6: “*My bias is that in a younger person,* [intervention 1] *probably is a better treatment … Rather than putting them into trial, I think what I’d like to do is give them* [intervention 1] *up-front.*”

While doctors continued to identify recruitment barriers as being organisational difficulties, the lack of patients fitting the RCT eligibility criteria, and patients expressing strong treatment preferences or rejecting randomisation, a careful analysis of the interview data indicated that these obstacles were not quite as straight-forward as they seemed. In the interviews, doctors readily expressed considerable discomfort and difficulty in relation to their roles as clinicians and recruiters, particularly around the eligibility of particular patients or groups, and their own treatment preferences, clinical instincts, and ability to feel uncertain about the most appropriate treatment. Many of these barriers seemed to originate from the doctors’ views or at least to be reinforced by their views. Their lack of equipoise for groups or individual patients contributed to the lack of eligible participants as they did not recruit those outside their comfort zone. If patients expressed the same preference for a treatment as the clinician, it was easy to allow them to have that treatment rather than offer recruitment to the RCT:

T6–D9: “*If you believe* [intervention 1] *is the best for them, you should say so … There’s somebody I saw recently … I actually took him off the trial. I said, ‘Well you’d be better off* [with intervention 1 rather than intervention 2]*’. So it’s a small number of people, but I hope I would offer them what I thought was the best treatment and take them out of the trial.*”

T6–D13: “*I believe that patients come through the door with preconceived ideas and in a lot of cases with their mind made up, and they’re checking that prejudice out, if you like, with the doctor when they come in.*”

T2–D2: “*I think there is a problem with equipoise … There are patients whom* [specialists] *regard as really high risk and they would be uncomfortable about putting them in, so they don’t actually.*”

Doctors were thus able to express commitment to the RCT because of the need to gather evidence but also considerable discomfort about particular aspects of recruitment, only rarely linking their views to recruitment problems.

### 2. Nurses’ roles

Nurses were employed as the primary recruiters in T3 and in the main recruitment phase of T6, and were closely involved in recruitment in the other RCTs as network nurses (T1, T2, T7) or care co-ordinators (T5). They discussed their roles in great detail, identifying three major aspects: caring clinical nurse, patient advocate, and recruiter/scientist. All the nurses perceived potential conflicts between these roles, particularly on the boundary between recruiter and caring nurse. Only a very small number thought their caring and recruiting roles could be complementary, but even they struggled with how to accommodate advocacy:

T7–N2: “*I hope that one complements the other really, rather than be conflicting. If it’s a study that I’m involved in then I think my background as a nurse would be – I would be there in a supportive sort of role, and also to be able to put something across to a patient where I felt that they didn’t feel intimidated, that they didn’t feel that they had to go into something, that it was, you know, really their choice to do that. I mean if you’re thinking about being an advocate, I don’t think that as a research nurse I can be.*”

The most common and powerful view that emerged was how concerned nurses were about the conflicts they perceived between caring for patients and recruiting them to RCTs. The boundary between research and clinical activity was a particular source of anxiety, and for those relatively new to research, adapting to a combined role was seen to be very challenging because of the differences they perceived in their status, employment, and the purpose of their activity:

T3–N1: “*I had to learn to adjust to quite a different role where actually I very much had to be invited to go into their family rather than being the person who’s going to go there and help them because although there is an element of that to my job, it’s not my main job. My main job is the research and to recruit. We’re not in uniform, because our first role is not as one of a nurse, it’s a researcher and I’m not employed as a nurse, I’m employed as a research associate by* [University] *… How will I know when I should be a nurse and when I should be a researcher? It’s really hard and there’ve been a couple of times when I’ve got a bit confused about that and thankfully it hasn’t led to anything drastic.*”

T2–N3: “*I haven’t had a real conflict yet, but I can imagine that sometimes it would be … You know, how many times do you pursue them, how many times do you say? And there is the bottom line that they want us to recruit people to these trials, don’t they? Obviously, in order for them to be a success, so you do feel, yes I do sometimes feel like a salesman.*”

When asked directly, most of the nurses stated that they enjoyed their roles as recruiters and their involvement in the RCTs, but as they discussed these issues in more detail in the interviews, it was clear that many of them wanted to define their position as caring clinical nurses, and they defended that role tenaciously. The primacy of their clinical/caring role was often asserted strongly, sometimes defensively, and many ensured their clinical role took precedence over the RCT:

T6–N3–G: “*I just tell them that they’re in control whatever they decide to do. If they decide to randomise, fine; if they reject the randomised treatment, fine; if they decide that they want the randomised treatment – whatever they choose to do as long as it’s an informed choice and I am satisfied it’s an informed choice, it’s entirely up to them. They’re in control of the whole process … We are nurses, we are here for them … We will support them through it because they are our priority. They come first, last and always. And that doesn’t always fit well with sort of, academics, but that is what we’re here for.*”

T2–N2: “*I always have the patient’s best interests at heart at the expense of the research. I’m not here to recruit patients onto trials and I always say to them, ‘I don’t mind what you do, the decision is yours, and if you’re not happy to do the trial, don’t do it.’ I would never recruit anyone that I didn’t feel was happy. I’d never talk them into doing it because that’s not – I am a nurse first and foremost.*”

The role as a caring clinical nurse was contrasted with the (lesser, rejected) role of researcher:

T6–N6–G: “*I don’t think we ever think of ourselves as being a researcher.*”

Interviewer: “*So – you always think of yourselves as nurses?*”

All: “*Yes.*”

T6–N1–G: “*Nurses carrying out research, but not researchers.*” 

Nurses had considerable influence in these RCTs over which patients were informed about the RCT and how it was presented to them. They strongly defended their professional right to make decisions about these things, but as they recounted decisions they had made, they sometimes expressed strong personal views about particular types of patients, the RCT design, and its interventions. Most assumed they could use their clinical judgment to decide who to approach about the RCT and that they would be able to do this, and recruit patients to the RCT, without transmitting their views:

T2–N2: “[Patients] *need to have great trust in their* [doctor]*, you know, that’s what I say to them. If you’re happy with your* [doctor]*, OK. If you’re not, it’s probably not the trial for them to go into … I know I must not have opinions on the studies and mustn’t influence them but for their own peace of mind I think they do need to have trust in their* [doctor] *just in case they’re randomised to* [intervention 1].”

T7–N3: “*You can tell when you’re speaking to patients, if they absolutely want the* [organ] *removed, you’re not going to be able to sway them, and I don’t think it would be ethical to try to be honest … I think if it was me in the situation or my dad in the situation I think I’d … rather* [have intervention 1] *… I keep that inside my head … If someone asked me, what would I do, if I can get out of answering the question, I would, but I’d never lie to somebody.*”

Some nurses were much more open with the interviewer about how they could and did make decisions that affected recruitment. In terms of their ability to decide who would or would not be informed about the RCT, two themes emerged.

#### Using discretion over whom to approach

Some nurses defended their right to use their discretion, based on what they believed to be their clinical/caring or advocacy role, and did so assertively. There may have been good clinical reasons for decisions, but in others, it appeared to be their own discomfort with the process of recruitment, or aspects of the RCT, and even prejudiced views about particular groups or individuals:

T2–N3: “*He was a very indecisive man generally, very indecisive, and in the end he literally just couldn’t make up his mind what he wanted to do … That’s a nursing decision if you like. You get to know what people are like and whether some – as I say like that indecisive man, he wouldn’t have coped with* [having intervention 1]*. You just know he wouldn’t because it’s his nature.*”

Interviewer: “*Does that mean that you actually make the final eligibility decision when you see the patient? You might decide not to approach somebody, at that point?*”

T2–N3: “*Yes I think that would be fair to say. Yes.*”

T7–N3: “*There was a patient who was young and he was clinically eligible, and basically he wasn’t very intelligent, and he would’ve been very easy to coerce to get on the trial. He would’ve gone on it, but I didn’t feel he was able to make a rational fully-informed decision, so I made the decision that he wasn’t going on the trial.*”

T3–N2: “*Sometimes you have to use your discretion about who you approach and not. You can see that a* [potential participant] *is there and she’s obviously very stressed, she’s got a screaming child and you kind of think ‘I don’t think she’s going to take part in the study anyway ‘cause her stress levels are just too high.’ So you don’t.*”

#### Apologising for ‘bothering’ potential participants and avoiding recruitment

Some nurses found the decisions about who to approach about the RCT so uncomfortable that they became very hesitant. They became awkward and apologetic about approaching potentially eligible patients, or found reasons to avoid this discomfort:

T3–N1: “*Recruiting* [participants] *to a study when they’re ill, that’s the most difficult time to ask a* [participant] *to take part in something. And that’s quite difficult and at times I almost feel apologetic, I think is the word, for approaching them … At the beginning, there were times where I felt I don’t know whether to approach them. I don’t know why, maybe it’s stereotyping, maybe it’s the way they look, the way they dress. I don’t know what else might be coming in and affecting me but I’m thinking ‘I’m not sure whether to approach them because I’m worried about being rejected, being told to go away’ … It was quite difficult to approach families that you thought didn’t speak English and that took quite a lot of courage on my part because you’re in the middle of a health centre, where everyone’s listening to you, and there’s nothing worse than actually not being understood or feeling that they’re not understanding you or that you’re causing them concern as well so I found that quite difficult … There was actually a* [potential participant] *I didn’t approach last week in* [location]*. Her attitude at the desk was very aggressive … and I thought I’m just not going there. No, so I didn’t.*”

Nurses had considerable opportunities to inform patients about ways to overcome organisational issues, but they tended to just reinforce the difficulties, or transmit their own views:

T2–N1: “*I can’t imagine having been already told by my doctor that this is what they are going to do for me and then be told by somebody else that hang on a sec, we are going to cancel all that and you could then potentially have* [another intervention] *than had already been scheduled. I mean I think that is a massive stumbling block.*”

T1–N1–G: “*I haven’t recruited anyone to the trial. Of the fifteen patients which have been registered, six of them came to us from outside* [city] *… It wasn’t in my conversation to ask these patients to be randomised because they were coming for* [intervention 1]*.*”

T1-N2-G: “*My patients are right smack bang in the middle of a socially deprived area, very socially deprived, not amazingly well educated – not to say that everyone’s stupid because you get some who are quite well educated – but the majority of them aren’t and they’re just, so you know, they’ve just got these preconceived ideas and they don’t want to think about it. Cancer means they’re dead. That’s it … They don’t want to go to* [name of] *hospital because they think once they go … there, they’ll die because that’s where you go to die. So you’re asking them to go there to have* [intervention 1]*, but they don’t want to go there because that means they’re going to die.*”

There were particular difficulties in T5 where the nurses and researcher struggled to understand some of the basic principles of the RCT design, and where they also had strong personal treatment preferences:

T5–N3: “*What is an RCT, sorry? …* [Intervention 1] *is better and the reason why I believe that is that it provides the extra support that the* [patient] *requires.*”

T5–N4–G: “*A couple of our* [patients] *have gone along and then they’ve got allocated to the control arm … I know that it’s a randomised trial, I’ve done research but from a clinician’s point of view – and this is what we’ve always quite openly sort of communicated to the* [T5] *project and this is why sometimes we have had difficulties – because we’d like to sort of say ‘right okay can we refer to the* [intervention arm]*?’*”

T5–N1: “*I feel a bit uncomfortable … The fact that I want them to get the intervention group, I’m sure that’s coming through. I was sure that I was sometimes trying to downplay that but I’m sure that my voice changes, there’s a lilt* [laughs]*.*”

Nurses identified recruitment barriers as being organisational difficulties, the lack of patients fitting the RCT eligibility criteria, and patients expressing strong treatment preferences or rejecting randomisation. Analysis of the interview data revealed, however, the nurses’ considerable discomfort and difficulty in relation to their roles, particularly in terms of which patients they approached to discuss recruitment to the RCT, a lack of knowledge for some about the RCT design, and their own views about logistical issues, treatment preferences, and patient characteristics. These views were expressed similarly by those employed by the wider networks and in the specific RCTs. It was also clear that many found their role as a recruiter conflicted with their perception of their clinical, caring, and advocacy responsibilities, leading to considerable discomfort and some clear instances of avoided recruitment. As with the doctors, above, some of the recruitment barriers could thus be seen to be originating from, or being reinforced by, the nurses’ views. Their unwillingness to approach individual or groups of patients contributed to the lack of eligible participants. If patients expressed a preference for treatment that accorded with theirs or fitted their view of the logistical difficulties, it was easy to allow them to have that treatment rather than offer recruitment to the RCT.

### Reconsidering barriers to recruitment: the need for support and training for recruiters

The synthesis showed that the clear obstacles and hidden challenges were found to some degree in all these RCTs. In T6, with its dedicated research staff, a programme of training and support was put in place for recruiters and integrated into the recruitment activity [8]. A more limited version of this intervention was included in the other RCTs because of funding and time constraints, and difficulties with engaging with the non-RCT funded network nurses [13-15]. A number of key priorities for training and support for recruiters emerged from the synthesis, including:

•For all recruiters: training and support to ensure they understand and can communicate to patients the key aspects of the RCT design, and can learn how to gently elicit and explore patients’ perceptions of intervention preferences and logistical challenges rather than reflecting their own views.

•For doctors: support to ensure they are committed to the RCT design and comfortable with the eligibility criteria and all intervention options.

•For nurses: support in relation to perceived conflicts in their roles; training to ensure they do not stray from clear clinical reasons for not approaching potential participants, and training to enable them to be comfortable with approaching all eligible patients.

The insights from the synthesis about the recruitment process and the roles of recruiters, as well as these training priorities, will be added to an updated version of the recruitment intervention in due course.

## Discussion

This study has provided a clear understanding of the perspectives of clinical staff attempting to recruit patients to pragmatic complex intervention RCTs. The findings highlight the fragility of the process of recruitment, and the overt/covert, witting/unwitting influences that recruiters can have on the success or otherwise of recruitment to RCTs. Recruiters readily identified organisational difficulties, fewer than expected eligible patients and strong treatment preferences expressed by patients as the key barriers inhibiting recruitment, but were seemingly unaware of their own influences on these obstacles. A key finding from this research was how much discomfort and emotion recruiters expressed about their research and clinical roles while also indicating strong commitment to the RCT and research in general. Doctors emphasised the complementarity of their roles as scientists and clinicians, but were uncomfortable about particular groups or individual patients, clashes with their clinical judgments, and their own preferences for particular interventions. Nurses identified conflicts arising from their roles as caring nurses, patient advocates, and (somewhat denigrated) recruiters/researchers. They were uncomfortable and defensive about the decisions they made about whom to approach about the RCT, sometimes avoiding ‘bothering’ patients, making ostensibly ‘clinical’ decisions that strayed into personal views, even prejudices, and tended to transmit their own views about treatments and organisational difficulties.

Recruitment was very difficult in these RCTs, as it is in many. There were many examples in the interviews of how recruiters privileged their clinical roles and judgments over recruitment, leading to large numbers of patients who were technically eligible for the RCT but who were not informed about it, and many others who were subtly (or not so subtly) manoeuvred towards a particular intervention for reasons that might have been clinically appropriate but more often reflected the recruiters’ views. It seems plausible that these findings would also be present in other pragmatic RCTs. They are novel because they help to explain why problems with recruitment can persist even when CIs work hard to resolve organisational problems that appear to be the source of the difficulties [2-6]. Recruiters in these RCTs often expressed strong commitment to the RCTs, but they found the issues raised by actually recruiting individual patients so discomforting that they found ways to avoid it, keeping their discomforts away from colleagues or RCT leaders.

Patients’ treatment preferences were identified as a major reason for poor recruitment in all these RCTs, as they were in other studies [16,17]. The nuanced analysis in this study suggested that some (perhaps many) of these preferences might actually be contributed to by the recruiters’ own views. A recent study of 93 consecutive recruitment appointments in T6 showed that patients’ treatment preferences ranged on a continuum from a strong desire to receive a treatment to a mild wish, based on a range of detailed or sparse, accurate or inaccurate information, gleaned from a variety of formal and informal sources [18]. When nurse recruiters carefully elicited and explored these preferences and provided detailed information about the RCT and its interventions, only a minority of patients remained committed to their initial preference, and the majority (75 % of those who had originally expressed a preference) became open to other options and 80 % of these then consented to randomisation [18]. The evidence from this synthesis was that recruiters often expected patients to have preferences, and when they heard preferences that seemed to make sense because they accorded with their own views, it was easy to accept them on face value, without further exploration. Some recruiters justified this by stating that it would be coercive to pursue things further; the balance between coercion and accepting stated preferences without checking they are based on accurate information would benefit from further investigation.

Doctors’ accounts showed that they were particularly uncomfortable with the formal RCT inclusion/exclusion criteria, reflecting concerns about ‘equipoise’. The literature on equipoise in relation to RCTs has largely been theoretical, focusing on its use as the ethical justification for randomisation [19-21]. The concept has been strongly criticised for its lack of relevance to contemporary healthcare [22,23], but there is remarkably little empirical research investigating how doctors talk about or operationalise equipoise during RCT recruitment (see, for an exception, [24]). In this study, most of the doctors could explain the concept in a scientific sense and their reliance on a sense of uncertainty as a community of experts, but they often went on to indicate their lack of uncertainty (certainty) in relation to specific patients or subgroups of patients or particular interventions. As a result, they made their own decisions about whether to apply the criteria fully or not, but did not raise these issues with the RCT CIs. This ‘variable enactment of the protocol’ was also found in another qualitative study of clinical centres in a single RCT [25], and is probably widespread.

Nurses were particularly vexed by the conflicts they perceived in their roles. This has been found in other small-scale studies, particularly for nurses working on the boundary between clinical work and research [26-28], but the influence on recruitment has not been shown previously. Most nurses, whether employed by central networks or by specific RCTs, saw research roles as of lower status and were clear that their clinical role would always come first ‘at the expense of the research’. Their discomfort was covertly expressed through their concerns about ‘not bothering’ patients with information about the RCT, or making ‘clinical’ judgments. For most, the discomfort caused anxiety; for a small number this anxiety became quite severe. Ultimately, however, their views and actions meant that many patients were not approached for recruitment, or were influenced in their decisions about treatments outside the RCT. It will be interesting to see if this changes with the introduction of initiatives such as the UK’s ‘OK to ask’ campaign encouraging patients to request research participation [29].

These issues have relevance for the increasing number of RCTs that are currently being launched. RCTs are needed so that only the most effective and cost-effective healthcare interventions are used, but they are extremely expensive to undertake. Failure to complete recruitment wastes considerable resources, and fears of recruitment difficulties mean that some key healthcare questions are avoided. These findings suggest that training and support for recruiters based on integrated qualitative studies to investigate the enacted recruitment process, might help with completing recruitment (as it did in T6, and to some degree in T3 and T5), or in clearly identifying the issues that led to the closure of T1 and T7.

The findings are also important in the context of the increasingly important contribution that nurses make to RCT recruitment. Over the past decade, nurses have become the main workforce in network organisations such as the UK NCRN, which co-ordinates recruitment for cancer RCTs, including T1, T2, and T7 [29]. Many have taken on this role without much formal training – often simple courses in ‘good clinical practice’ , or occasionally more advanced specialist courses [30]. As recruitment to RCTs is increasingly placed in the hands of nurses, if the views expressed here have currency elsewhere, then considerable opportunities for RCT recruitment are not being taken. Nurses had considerable power to decide which patients would be given information about the RCT, justified by being the patient’s advocate. Advocacy is a relatively new addition to nursing, and while it is often promoted as a moral imperative, it is fraught with dilemmas [31,32]. It will be important for further research to examine the role of advocacy and clinical decision-making in nurse-led recruitment. It was remarkable how little insight nurses seemed to have about the judgments they were making, and how little evidence there was that they had taken time to check the veracity of their opinions with patients. Training and support in T6 enabled nurses to achieve very high rates of recruitment and informed consent [8]. There was evidence in this synthesis that the nurses still continued to exhibit some concerns about their roles, but they had access to support that mitigated their anxiety. These nurses, and those in T3, were easiest to train because they were employed by the RCTs and time was dedicated to it by the CI; it was very difficult to meet with groups of nurses who were employed by the national networks such as the NIHR NCRN because they were responsible for recruitment to many RCTs and their supervisors were unwilling for them to devote time to particular RCTs. The intensive training and support in T6 may not always be possible, but this synthesis has emphasized the need for standard and continuing training for all nurse recruiters, to include aspects of RCTs, roles, and social awareness, and not just good clinical practice. It has also been suggested that standard and continuing education is needed for physicians about how participation in RCTs varies from standard clinical practice [33]. The evidence from this synthesis suggests that all recruiters (physicians, surgeons, nurses, and other professionals) would benefit from training incorporating basic and advanced details of the RCT design and how to present treatments and randomisation fairly and clearly.

### Strengths and limitations

This study is the first detailed and wide ranging exploration of the perspective of recruiters involved in recruitment in several (six) pragmatic RCTs underway with recruitment difficulties. A strength of the study was in the use of qualitative methods to understand the perspectives of those actively engaged in recruitment, producing a detailed and rich synthesis that allowed a nuanced understanding of the processes underlying recruitment in each RCT across a range of clinical interventions, recruitment methods, and contexts (Table 1). Qualitative research methods are particularly suitable for gaining an understanding of complex issues and systems and the perspectives of particular individuals and groups.

The study has a number of limitations. The RCTs were included pragmatically because they included or added a qualitative study with the aim of improving their recruitment, and so might be different from other RCTs. There was diversity in the RCTs in terms of clinical contexts, but four involved oncology and three surgery. There were differences in the numbers and characteristics of those who were interviewed in the RCTs and interviews and group discussions were analysed pragmatically – a function of the different management and recruitment arrangements. It is likely that only those most willing to speak about the RCT and recruitment issues agreed to be interviewed, and so there could be different views among other recruiters. The data were collected across quite a long time period because of the reliance on the funding of the qualitative studies. Accumulating evidence of this sort across complex studies is difficult and this is the largest synthesis to date. The consistency of findings over time suggests that the insights are robust, but it cannot be ruled out that other or more recent RCTs recruit better (although recruitment problems remain the key challenge to RCTs today). It is important to acknowledge that the RCTs here were all having recruitment difficulties. Others may have ‘quick fix’ issues that simple interventions such as telephone reminders, etc., may solve [2,6]. However, for RCTs where the topic is controversial or the evidence is contested, or interventions are very different (including ‘no’ or ‘control’ intervention arms), it is likely that patients and clinicians will have strong views or preferences, and it may be that the findings have most relevance for these most ‘challenging’ RCTs.

## Conclusions

This study suggests that there are tremendous opportunities to increase levels of RCT recruitment if some of the basic and discomforting issues that trouble recruiters can be explored and training put in place to support them when undertaking this fragile activity. The study has provided the insight that, while trial recruitment (randomisation) is a simple event, it occurs at the end of a long chain of activities leading from the design of the RCT, through the operationalization of the protocol in clinical settings, to the presentation (or not) of the RCT to patients who have been investigated for eligibility. Recruitment difficulties are often seen to arise from organisational issues, patient shortages, and strong intervention preferences, but, as this synthesis has shown, many of these issues probably reflect underlying issues among recruiters in terms of knowledge and views about evidence, equipoise, RCT design, role conflicts, specialty interests, and particular personal preferences. The synthesis showed that clear obstacles and hidden challenges were found to some degree in all these RCTs, but their relative importance varied in each case, underlining the particular value of the qualitative research in understanding specific contexts, and also the need for targeted and specific (as well as generic) training.

Recruitment is a difficult activity because it disrupts the usual doctor- (or nurse-) patient relationship. Training and support are required to ensure recruiters understand the principles of RCTs and can explain the rationale for the RCT to potential participants, have commitment to the RCT, are confident in admitting uncertainty, are willing to approach all eligible patients, can elicit patient preferences and explore underlying reasons for them, and provide accurate information. It may be that this is not an activity that all doctors and nurses can or should undertake, and those with very strong views about treatments or stereotyped views should clearly not undertake recruitment. Those leading RCTs with recruitment difficulties will need to consider how the ‘clear obstacles’ and ‘hidden challenges’ likely to be operating can be addressed. The support and training needs for doctors and nurses are different but individual practitioners will need to acknowledge the anxiety and emotion they experience and accept training and support if we are to create a more resilient recruitment process that engages patients and recruiters more effectively.

### Additional information

Approvals for research were given for RCTs T1, T2, T3, and T5 through the MRC Quartet study by the NHS Research Ethics Committee, Leeds West (07/02/2005; ref.: 04/Q1205/179); for T6 through the ProtecT study, by Trent Multicentre Research Ethics Committee (ref.: 01/4/025); and for T7 through SPARE RCT, by South East Research Ethics Committee (ref.: 06/MRE01/95).

## Abbreviations

CI: Chief investigator; NCRN: National Cancer Research Network; RCT: Randomised controlled trial; TMG: Trial management group.

## Competing interests

The authors declare that they have no competing interests.

## Authors’ contributions

JLD conceived the study, obtained funding, and carried out the analytical synthesis. SP, IdS and MT collected, analysed and reported data relating to particular RCTs. JLD wrote the first draft of the manuscript. All authors contributed to the interpretation of the data and critically reviewed the intellectual content of the final manuscript. All authors read and approved the final manuscript.
